# Diabetic Retinopathy: A Pharmacological Consideration

**DOI:** 10.7759/cureus.46842

**Published:** 2023-10-11

**Authors:** Saket Y Maheshwari, Sunil Kumar, Arya Harshyt Sinha, Mayank Kumar

**Affiliations:** 1 Medicine, Jawaharlal Nehru Medical College, Datta Meghe Institute of Higher Education and Research, Wardha, IND; 2 Anatomy, Jawaharlal Nehru Medical College, Datta Meghe Institute of Higher Education and Research, Wardha, IND; 3 Community Medicine, Jawaharlal Nehru Medical College, Datta Meghe Institute of Higher Education and Research, Wardha, IND

**Keywords:** diabetic macular edema, blindness, vision loss, diabetic retinopathy, diabetes mellitus

## Abstract

Diabetes mellitus (DM) has become a worldwide problem, endangering the well-being of people. This issue is further aggravated by the increased fatty content in the diet of most of the Indian population. It is a preeminent source of the genesis of morbidity in the citizens of any given continent, including both new-world countries and old ones too. A major stumbling block that diabetes creates in the healthy living of any of its sufferers is a complication called diabetic retinopathy (DR), which, in its most elementary and perspicuous form, refers to damage to the blood vessels in the retina of the human eye that occurs as a result of high serum glucose levels. DR can have many symptoms, including obscure and blurred vision, trouble observing and distinguishing various colors, and eye floaters. One of the most significant reasons for the manifestation of new cases of complete blindness may be attributed to DR. The appearance of lesions in the body's small blood vessels forms the basis of retinopathic detection. The currently accepted approach for the prevention and cure of this ailment targets deterring the microvascular complexities through medicinal agents that are placed directly into the vitreous space, photocoagulation through laser medium (visual perceptivity is balanced), and some other surgeries related to the vitreous chamber. Anti-vascular endothelial growth factor (anti-VEGF) therapy provided to the patient by intravitreal route is, at present, the most crucial process for curing the sufferer of the given illness, as it can result in optical advancement with decreased unfavorable effects.

## Introduction and background

Diabetic retinopathy (DR) stands as a significant global concern in ophthalmology, affecting millions of individuals worldwide and presenting a substantial public health challenge. Characterized by the weakening of blood vessels within the eye, leading to blood leakage and severe vision impairment [1], DR's prevalence is alarming, particularly in diabetic populations, with a significant percentage of individuals facing the risk of vision impairment or blindness due to this condition. Recent statistics from credible sources highlight the scale of this issue. For instance, as of 2015, it is estimated that over 90 million people worldwide are affected by DR [2]. These statistics underscore the urgent need for comprehensive research and intervention in the field of DR.

DR is conventionally categorized into two key stages: non-proliferative DR (NPDR) and proliferative DR (PDR). NPDR represents the initial phase, characterized by heightened capillary permeability and capillary obstruction [3]. Remarkably, even asymptomatic individuals can exhibit early retinal abnormalities, including microaneurysms and the leakage of blood and fluid from ruptured capillaries, observable through fundus photography.

As DR advances, it transitions to the more advanced stage of PDR, marked by the development of abnormal retinal blood vessels. PDR carries an elevated risk of acute vision impairment, especially when new aberrant arteries extend into the vitreous or tractional retinal disengagement occurs [3].

One of the most devastating consequences of DR is diabetic macular edema (DME), often resulting in complete vision loss. DME is characterized by macular swelling due to fluid accumulation beneath and within the retinal layers, primarily triggered by the breakdown of the blood-retinal barrier (BRB) [4]. This review aims to delve into the pharmacological considerations for managing DR, focusing on potential treatments and interventions to mitigate its impact on patients' vision and quality of life.

## Review

Methodology

Literature Search

We utilized various databases such as PubMed, the Cochrane Library, and Google Scholar. Our search included specific keywords relevant to our study, including "diabetic retinopathy", "treatment", "newer drugs", "pathology", "surgeries", and "prevalence". We also manually searched the reference lists of relevant articles to identify additional studies.

Inclusion and Exclusion Criteria

Incorporated in our research were studies that explored the various aspects of DR, such as pathology, epidemiology, diagnosis, treatment, and prevention. However, we opted to exclude non-English studies and those that did not undergo the peer review process.

Data Extraction and Synthesis

In order to conduct our analysis, we obtained information from each study that was included in the research. To evaluate this collected information effectively, we employed a narrative approach, which allowed us to provide an overview while highlighting important and current details.

Data Analysis

**Figure 1:**
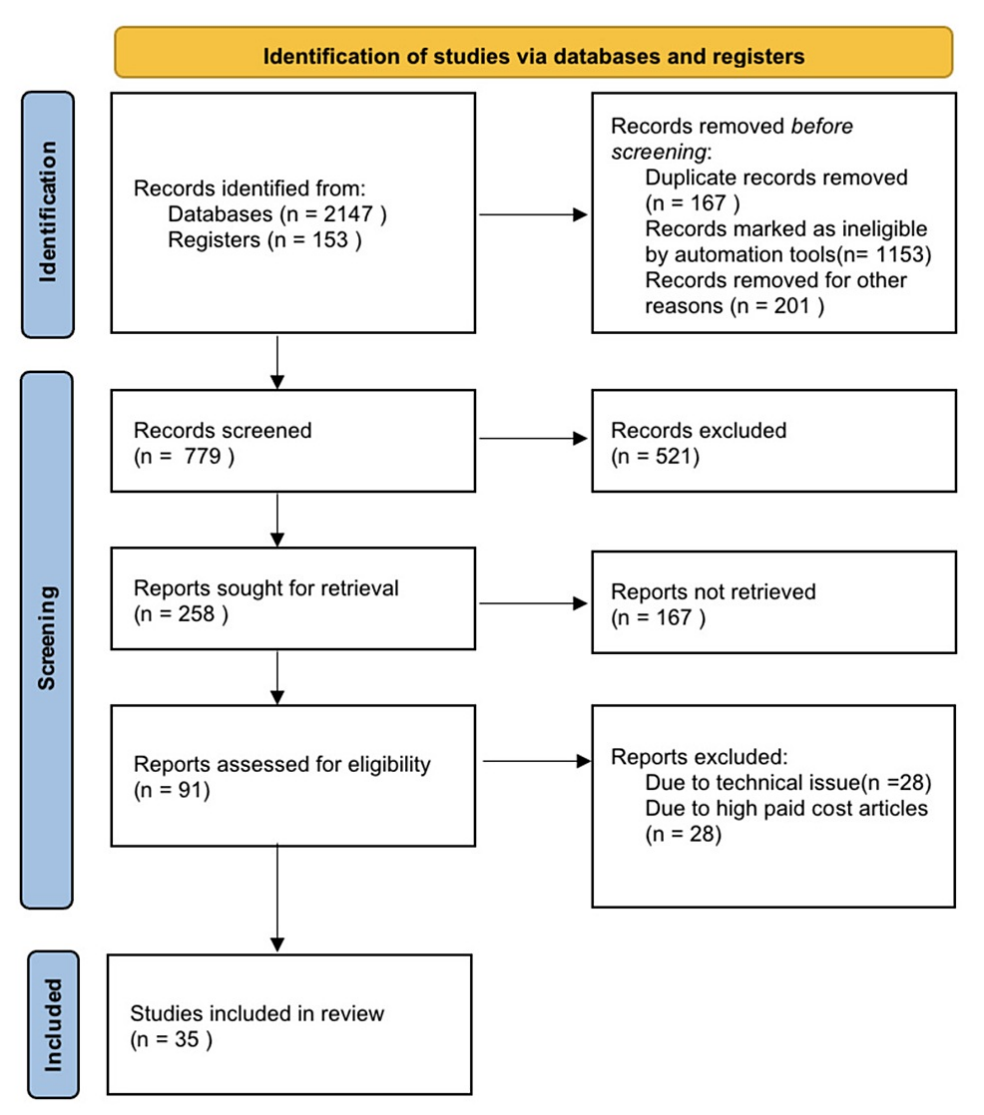
Search strategy utilized for this review

In our analysis, we employed a qualitative method to examine the data. We focused on identifying recurring themes and patterns that emerged from the studies included in our research. Furthermore, we utilized descriptive statistics as a means of succinctly summarizing the findings obtained. Figure 1 shows the search strategy utilized.

Pathology

Retinal Microvasculopathy and High Blood Sugar

Hyperglycemia is thought to impact the development of ocular microcirculatory injury tremendously. The polyol route, the buildup of advanced glycation end products (AGEs), the protein kinase C (PKC) pathway, and the hexosamine biosynthetic pathway are all consistently linked to and associated with metabolic derangement and endothelial dysfunction [1].

Inflammation: The inflammatory response is a crucial step in the development of DR. Across diabetic experimental animals and patients, chronically low inflammation in the body has been observed at various stages of the disease. In the preliminary phase of DR, leukostasis has already been identified as a meaningful step [3].

Neurodegeneration of the retina: Initially, in the course of DR, visual degradation occurs. In hyperglycemic mice, cellular senescence of neuronal cells can be seen as soon as 30 days after diabetes onset [4].

Diagnosis

A dilated eye exam is the perfect and reliable way to detect DR. The sooner the detection, the better the prognosis of this "otherwise dangerous" plight will be [5]. During this procedure, the mydriatics introduced into the human eyes expand the pupils to give the clinician a superior view of the ocular system. During the checkup, the ophthalmologist will also review the inside and outside for any latent issues that may have been the reason behind the development of blurred vision. A prominent process for diagnostic help is fluorescein angiography; fluorescein is administered intravenously into one arm following successful pupil dilation. This dye is then circulated into the vascular system in one's eyes, and photographs are obtained afterwards [6]. Blood vessels that are occluded, damaged, or oozing can be identified using these pictures. This contributes significantly to patients' favor regarding their health. The width of the retina is revealed by the bridge images produced by optical coherence tomography (OCT). This should assist in identifying how much liquid has seeped into the retinal tissue, if any at all. OCT tests could then be used to track and assess how promptly the regimen is functioning [7]. Table 1 shows various diagnostic criteria of DR.

**Table 1:** Diagnosis of DR DR: diabetic retinopathy

Diagnosis	Criteria
Background DR	Microaneurysm, hard exudate, retinal edema
Maculopathy	Retinal edema/thickening at macular region
Pre-proliferative	Cotton wool spots, vascular abnormalities, venous beading
Proliferative DR	Neovascularization at the disc, neovascularization elsewhere
Advanced eye disorder	Vitreous hemorrhage, pre-retinal fibrosis, tractional retinal detachment

Treatment

DR care depends on several variables, such as the complexity of the affliction and the immune status of the victim. The precision with which the chosen doctor operates on his subject is also essential in deciding the subject's fate. Throughout the beginning phases of the process, a practitioner may choose to keep a watchful eye on the patient's eyes without meddling. The term for this strategy is "vigilant waiting". This is an excellent approach to substantiating the gravity of ill health and acting accordingly [8-11].

Laser Surgical Intervention

To assist in the shutting off of bleeding veins and arteries, beam ablation may be used. This can help minimize retinal edema. Capillaries can be shrunk and prevented from sprouting again via corrective surgery. Occasionally, well over one measure is necessary, as this might not be enough for the betterment of the sufferer [12-15].

Photocoagulation

A considerable halt or decrease in the loss of plasma and fluids from the eyes may be pulled off by a procedure commonly referred to as focal laser therapy. Laser burning addresses leakage from aberrant vasculature during the treatment [8]. A solitary round of focused laser treatments is usually performed in the doctor's facility or an eye clinic. When patients have macular edema and have had eye strain preceding the operation, the standard of care may never restore one's eyesight. Still, it might lessen the risk of the visual edema deteriorating [16-19].

Photocoagulation of the Entire Retina

The malformed blood vessels can be shrunk using this phototherapy, also called scattered laser repair. The portions of the retina farthest from the center are fixed with dispersed laser burning as part of the process. The burns fade and ruin the faulty, budding circulatory tubes. Before the introduction of anti-vascular endothelial growth factor (anti-VEGF) medication, laser photocoagulation was the holy grail for the rehabilitation of DME and DR [20, 21]. In the Early Treatment Diabetic Retinopathy Study (ETDRS), central focus macular laser treatments were demonstrated to adequately decrease macular edema and minimize the likelihood of intermediate vision deficit by a massive 50% range [22].

Modern laser techniques: Systems are currently being developed to create new laser methods to lessen negative impacts. PASCAL (OptiMedica Corporation, Santa Clara, CA) (pattern scanning laser) is a novel technique for treating DME [23]. It decreases laser-induced damage to the eye by allowing for highly accurate beam regulation and faster recovery times [24].

Treatment aimed against the genesis of new blood vessels

Anti-VEGF Medications

The introduction of anti-VEGF medicine has reshaped and transformed how DR is treated. The generation of this solution has helped humanity avoid the instigation of blindness to an extent that was practically impossible for the earlier generations to even imagine [25]. Pegaptanib, ranibizumab, aflibercept, and the off-label intravitreal bevacizumab are several lauded anti-VEGF pharmaceuticals that have been studied and researched in drug trials for DR therapy [8, 26-30]. Ranibizumab has received the most detailed and extensive testing of these agents. It was the prototype drug during the initial exploration phase of this method. They aid in the prevention of fresh blood vessel formation and the reduction of fluid accumulation. An external anesthetic is used to administer these medications. For up to 24 hours following the infusion, moderate discomfort such as searing and ripping pain, or soreness may occur. It is not necessary to view these modifications as extremely strange [10].

Vitrectomy 

The doctor might suggest a vitrectomy operation if the eyes suffer severe PDR. But it is to be remembered without any exception that the doctor should resort to this remedial method only in drastic cases. Intraocular gel and blood from broken vessels at the rear of the eye are removed by the doctor. Rays of light can now concentrate suitably on the retina once more. The retina's wound might even be excised. It is to be noted that this course of action has to be carried out with the utmost precaution. DR can be slowed significantly with intervention. However, this is hardly curative and the ill effects of this apathy may recur. Possible retinal degeneration and vision impairment are still conceivable, given that diabetes mellitus (DM) is a lifetime illness [31].

Some of the hazards associated with vitrectomy are more bleeding into the eye as a result of acquiring cataracts, moisture building up in the cornea due to retinal separation (the outer layer at the front of the eye), and a corneal infection.

Anti-inflammatory Therapy (AIT)

AIT is a type of treatment that is used to reduce inflammation. Intravitreal corticosteroids have now become more significant in managing DME, particularly when anti-VEGF therapy has failed (which rarely happens, but every possibility is to be considered) [11]. Numerous factors are thought to be involved in recurrent instances of DME and nonresponders to anti-VEGF therapy. As effective anti-inflammatory drugs, corticosteroids attack a wide range of molecules associated with the etiology of DME, namely, VEGF, tumor necrosis factor (TNF), pro-inflammatory cytokines, leukostasis, and tight junction peptide activation [12].

Nonsteroidal Anti-inflammatory Drugs (NSAIDs)

In the field of DR treatment, there is a growing interest in exploring the potential of NSAIDs to inhibit pro-inflammatory cytokines like interleukin-6 (IL-6). IL-6, a prominent cytokine frequently found in the vitreous of DR patients, has gained attention as a potential target for NSAID-mediated inhibition. To harness the anti-inflammatory potential of IL-6, researchers have developed specialized immunoglobulins tailored to counteract its effects. Two noteworthy examples are EBI-031, which directly targets IL-6, and tocilizumab, designed to inhibit the IL-6 receptor.

These innovative medications hold promise in mitigating the inflammatory processes associated with DR. By addressing the role of IL-6 in the disease's pathogenesis, these non-steroidal options offer an exciting avenue for improving the management and outcomes of DR, ultimately enhancing the quality of care for affected individuals [16].

Alpha-Lipoic Acid (ALA)

In the realm of DR treatment, ALA has emerged as a noteworthy contender, providing a glimmer of hope for individuals dealing with the complexities of both type 1 and type 2 diabetes. Research has unveiled a compelling association between ALA consumption and enhanced eyesight in diabetic patients, shedding light on its potential mechanisms of action and therapeutic effects. ALA, a natural antioxidant, wields its power by combating oxidative stress, a pivotal player in DR's pathogenesis. In diabetes, high blood sugar levels trigger the production of harmful free radicals within the retina, leading to cellular damage. ALA's potent antioxidant properties intercept these free radicals, neutralizing their detrimental effects and reducing oxidative stress. This mechanism not only helps protect retinal cells but also slows down the progression of DR [13].

Beyond its antioxidant prowess, ALA influences glucose metabolism and insulin sensitivity. It enhances the body's ability to efficiently utilize glucose, potentially assisting in glycemic control for diabetic patients. Improved insulin sensitivity further aids in glucose regulation, indirectly benefiting DR management. While ALA offers promising therapeutic potential, it's essential to consider potential adverse effects. Commonly reported side effects of ALA supplementation include gastrointestinal disturbances such as nausea and diarrhea. In some cases, skin rashes and allergic reactions have been observed.

In summary, ALA presents a multifaceted approach to DR treatment. Its antioxidant capabilities counteract oxidative stress, while its influence on glucose metabolism and insulin sensitivity contributes to better glycemic control. However, individuals considering ALA supplementation should be aware of potential adverse effects and consult with healthcare professionals for guidance. As research continues to unveil ALA's full potential, it stands as a valuable component in the comprehensive management of DR, offering the promise of improved vision and enhanced quality of life for those navigating this challenging condition [13].

Lutein

Time and again, the significance of lutein as the "vitamin of the human eye" has been reaffirmed through numerous studies. Lutein, a carotenoid, has garnered substantial attention for its potential in the treatment of DR. This organic compound's role in eye health is rooted in its ability to serve as a potent antioxidant and filter of harmful blue light. Emerging research has illuminated the promise of standard lutein treatment in the reversal of retinal abnormalities observed in diabetic mice. Furthermore, supplementation with lutein has demonstrated the potential to enhance functional status in individuals with NPDR. As ongoing studies delve deeper into its effects on human subjects, lutein stands as a compelling candidate for the comprehensive management of DR [13, 15].

The mechanisms underlying lutein's potential in DR treatment are multifaceted. As an antioxidant, it shields retinal cells from oxidative stress, a hallmark of DR pathogenesis. Additionally, its blue light-filtering properties protect the macula, thereby preserving visual function. Moreover, lutein's anti-inflammatory effects contribute to reducing the chronic inflammation often associated with DR progression. While its role in reversing retinal abnormalities in diabetic mice is encouraging, ongoing human trials aim to elucidate its precise impact and safety profile [13].

Despite its potential benefits, it's important to acknowledge the need for cautious evaluation of lutein's effects in human subjects, including any potential adverse effects. Comprehensive research will help ascertain its true therapeutic potential and optimal dosing strategies. In conclusion, lutein's role as the "vitamin of the human eye" holds promise in the treatment of DR. Its mechanisms of action, encompassing antioxidant, blue light protection, and anti-inflammatory properties, position it as a valuable component in DR management. As investigations continue to unfold its impact on human subjects, lutein remains an intriguing avenue in the pursuit of improved outcomes and enhanced quality of life for those grappling with this complex eye condition [12, 14].

ARA290

In the ongoing quest for effective treatments for DR, a phase 2 clinical trial is presently underway to assess the potential of ARA290 as a monotherapy for DME. ARA290, a peptide-based compound derived from erythropoietin's structural foundation, primarily recognized for its role in stimulating red blood cell production, sets itself apart by not participating in hematopoiesis [12]. The intriguing facet of ARA290 lies in its distinct mechanism of action. While it shares origins with erythropoietin, it steers clear of red blood cell generation. Instead, it exerts its therapeutic influence by engaging with the body's inherent repair mechanisms and anti-inflammatory pathways. ARA290 demonstrates promise in modulating the inflammatory response, reducing oxidative stress, and promoting tissue repair, all of which play critical roles in the pathogenesis of DR.

ARA290's distinctive attributes position it as a potential game-changer in the treatment landscape of DR. By addressing inflammation and oxidative stress, it holds the promise of retarding DR progression and managing the often-concomitant DME. Additionally, its non-impact on red blood cell production distinguishes ARA290 from erythropoietin-based therapies, sidestepping associated hematologic side effects. However, as with any therapeutic intervention, ARA290 is not without potential adverse effects, though it is generally considered safe. Some individuals may experience mild side effects, such as local irritation at the injection site. Rigorous monitoring throughout ongoing clinical trials will provide further insights into its safety profile. In conclusion, ARA290 represents a promising avenue in the comprehensive management of DR, harnessing its unique mechanism of action to target inflammation and promote tissue repair. As research and clinical trials continue to unravel its efficacy and safety profile, ARA290 holds the potential to augment the arsenal of treatments for DR, potentially enhancing the quality of life for those grappling with this intricate condition [12-14].

Ways to prevent DR vision loss

Individuals with diabetes are strongly advised to consult their family physician for guidance on maintaining optimal blood sugar levels to ensure good health. Prolonged hyperglycemia, characterized by persistently elevated blood sugar, can have adverse effects on retinal blood vessels, potentially leading to visual impairment. Therefore, seeking advice from a specialized healthcare provider is crucial when facing such complications. Patients can expect their healthcare provider to offer the most appropriate recommendations in such situations, as referenced in medical literature [32, 33].

It is essential for individuals to schedule regular dilated eye examinations with their ophthalmologist. This proactive approach enables the early detection of DR, even before noticeable visual disturbances occur. Prioritizing one's health is of paramount importance in managing diabetes. If any noticeable symptoms manifest in either or both eyes, it is imperative to promptly contact one's primary care physician. Swift treatment of type 2 diabetes is highly effective in preventing the development of debilitating health conditions, as corroborated by relevant studies [34-36].

## Conclusions

It's crucial to emphasize the use of medications as a central element in the comprehensive management of DR. Our extensive research strongly reinforces this essential point. Recent studies that have delved into specific pharmaceutical treatments have unveiled promising avenues for therapy. These treatments can help patients preserve their vision and slow down the progression of the disease, which is incredibly significant for their overall well-being. Understanding the full spectrum of available pharmacological options in the realm of DR is poised to make a significant impact on patient outcomes. This understanding not only benefits individuals by safeguarding their vision but also lessens the overall burden of this condition on both patients and healthcare systems. It's important to note that as ongoing research and advancements continue to shape the field of DR, the potential for further improvements in patient care and outcomes remains substantial. This progress is a testament to the continuous efforts to enhance our understanding and treatment of this condition.
